# Unraveling the Role of Polydopamines in Resistive Switching in Al/Polydopamine/Al Structure for Organic Resistive Random-Access Memory

**DOI:** 10.3390/polym14152995

**Published:** 2022-07-24

**Authors:** Jonghyeon Yun, Daewon Kim

**Affiliations:** 1 Department of Electronics and Information Convergence Engineering, Kyung Hee University, 1732 Deogyeong-daero, Giheung-gu, Yongin 17104, Korea; jonghyeon.yun@khu.ac.kr; 2 Institute for Wearable Convergence Electronics, Kyung Hee University, 1732 Deogyeong-daero, Giheung-gu, Yongin 17104, Korea; 3 Department of Electronic Engineering, Kyung Hee University, 1732 Deogyeong-daero, Giheung-gu, Yongin 17104, Korea

**Keywords:** organic resistance random-access memory, polydopamine, resistive switching, oxygen vacancy

## Abstract

In an era of rapidly evolving artificial intelligence and 5G communications technologies, massive data storage and processing are required for the real-time operation of digital processors in conventional wearable devices. However, classical von-Neumann architecture computers are limited by bottleneck-related issues. As a solution, resistive random-access memory (RRAM) devices are being considered as next generation in-memory computing devices. Among various materials, a polydopamine (PDA) is an attractive candidate for the fabrication of wearable and flexible RRAM devices. Herein, an aluminum/PDA/aluminum structure is proposed to investigate the influence of the PDA layer on resistive switching. The resistance-switching characteristics of an Al/PDA/Al structure are investigated by changing the PDA’s coating time and an on/off ratio of 2.48 × 10^3^ is recorded. X-ray photoelectron spectroscopy reveals the presence of an Al_2_O_3_ layer in Al/PDA/Al structure, and the contents of oxygen vacancies are changed according to PDA coating time. Conductive filaments in the PDA/Al structure are confirmed by conductive atomic-force microscopy. As an application, a flexible Al/PDA/Al structure is fabricated using polyethylene terephthalate substrate and its operation is successfully confirmed. These results describe the resistive-switching characteristics, including oxygen vacancies, of Al/PDA/Al structures and provide new ways of understanding the resistive-switching mechanism of PDA-based RRAM devices.

## 1. Introduction

Due to the rapid advancements in artificial intelligence and 5G communications technologies, various wearable devices have been proposed for real-time communication and healthcare applications with the potential to change lifestyles. The massive data storage and processing are required to operate the real-time digital processors embedded in these wearable devices. However, classical von-Neumann architecture computers suffer from bottlenecks during the exchange of information between data storage and processing devices, resulting in a reduction in their throughput [1,2,3]. These bottlenecks are crucial problems to be solved because fatal accidents can occur due to delays in real-time data processing. In-memory computing devices have attracted attention to handle the bottleneck issues in classic von-Neumann architectures. For example, resistive random-access memory [4,5,6,7], phase-change random-access memory [8,9,10], and spin-transfer-torque magneto-resistive random-access memory [11,12,13] have been proposed for in-memory computing devices.

Among these candidates, a resistive random-access memory (RRAM) device is considered as a promising next-generation in-memory computing device due to its simple metal-insulator-metal (MIM) structure [14,15,16], high switching speed [17,18], endurance [19,20], high density [21,22], excellent compatibility with complementary metal-oxide-semiconductor fabrication [23,24,25], and low power consumption [26,27]. The working mechanism of RRAM is based on resistive switching produced by a sudden change in its resistance. Although the precise working mechanism of the resistive switching is not yet known, one promising possible explanation is the repetitive creation and rupture of conductive filaments resulting from oxygen vacancies, which are formed by the migration of the oxygen ions in the insulator [4,28,29,30,31]. Hence, it is important to select a suitable material for the insulator to fabricate the RRAM devices. Various metal oxide-based RRAM devices have proposed because metal oxides possess sufficient oxygen to generate the required oxygen vacancies. However, conventional metal oxide-based RRAM devices suffer from drawbacks associated with inorganic materials such as poor flexibility and the need for a thermal process to synthesize metal oxides, indicating that metal oxide-based RRAMs are unsuitable for wearable devices. Hence, new materials are highly required.

Organic materials are attractive alternatives for fabricating flexible RRAMs because they are more flexible than inorganic materials, and the thermal process is not required to fabricate organic-based RRAM. Based on these advantages, various organic-based or organic-inorganic hybrid RRAMs have been proposed [32,33,34,35]. Among the organic materials, polydopamine (PDA) has attracted substantial attention because the PDA can be coated on the surface of almost all organic and inorganic materials due to the presence of functional catechol and amine groups. The PDA also exhibits strong adhesive properties and good natural biocompatibility [36,37]. However, a working mechanism has been veiled and uninvestigated for PDA-based RRAMs. To increase the applicability of the PDA’s organic RRAM, the working mechanism of a PDA-based RRAM should be addressed.

Herein, an aluminum/PDA/aluminum (Al/PDA/Al) structure is proposed to investigate the influence of a PDA layer on resistive switching. The resistance-switching characteristics of the Al/PDA/Al structure were confirmed by changing the PDA’s coating time, and the PDA’s coating time was optimized. As a result, an on/off ratio of 2.48 × 10^3^ was observed in Al/PDA/Al structures. The percentage distribution of voltage applied for the set-and-reset process in 196 (14 × 14) Al/PDA/Al structures was also investigated. Voltages for the set-and-reset process increased with PDA coating times. X-ray photoelectron spectroscopy (XPS) was used and a formation of an Al_2_O_3_ layer was confirmed. The occurrence of the oxygen vacancies was observed and investigated according to PDA’s coating time. The creation of the conductive filaments in the PDA/Al structure was also observed using conductive atomic force microscopy. These results reveal that the resistive switching in an Al/PDA/Al structure is based on the repetitive creation and rupture of the conductive filament depending on oxygen vacancies. The results of this study provide an improved understanding of PDA and PDA-based organic RRAM by unraveling its resistive-switching mechanism.

## 2. Materials and Methods

### 2.1. Fabrication of the Al/PDA/Al Structure

A silicon (100) wafer was prepared with the size of 2 cm × 2 cm. Using a shadow mask fabricated by Invar 36, the bottom aluminum electrode was deposited by a magnetron RF sputter at 150 W for 30 min in Ar gas at 30 sccm. After depositing the bottom Al electrode, a dispersion for PDA was prepared with a tris(hydroxymethyl)aminomethane (Tris, Sigma-Aldrich)-HCl buffer (pH 8.3~8.5). Then, a commercial-grade dopamine hydrochlorides (3 mg/mL) was added for polymerization. Ultrasonication was conducted to ensure a uniform PDA coating. After applying the coating process, the PDA-coated materials were cleaned with deionized water to remove the residues from the surface. Then, the PDA-coated materials were dried for 1 h in the shade at room temperature.

### 2.2. Characterization

A source meter (Keithley 4200A, Tektronix, Beaverton, OR, USA) and pulse generation module (4225-PMU, Tektronix, Beaverton, OR, USA) were utilized to measure the I-V characteristics of the Al/PDA/Al structure. A scanning microscope (MERLIN, Carl Zeiss, Jena, Germany) was used to investigate the cross section of the PDA/Al structure and an atomic microscope (XE7, Park Systems, Suwon, Korea) was used in order to confirm the conductive filament. X-ray photoelectron spectroscopy (K-Alpha, Thermo Electron, Waltham, MA, USA) was utilized to survey the spectrum of the surface of the PDA/Al.

## 3. Results and Discussion

**Figure 1:**
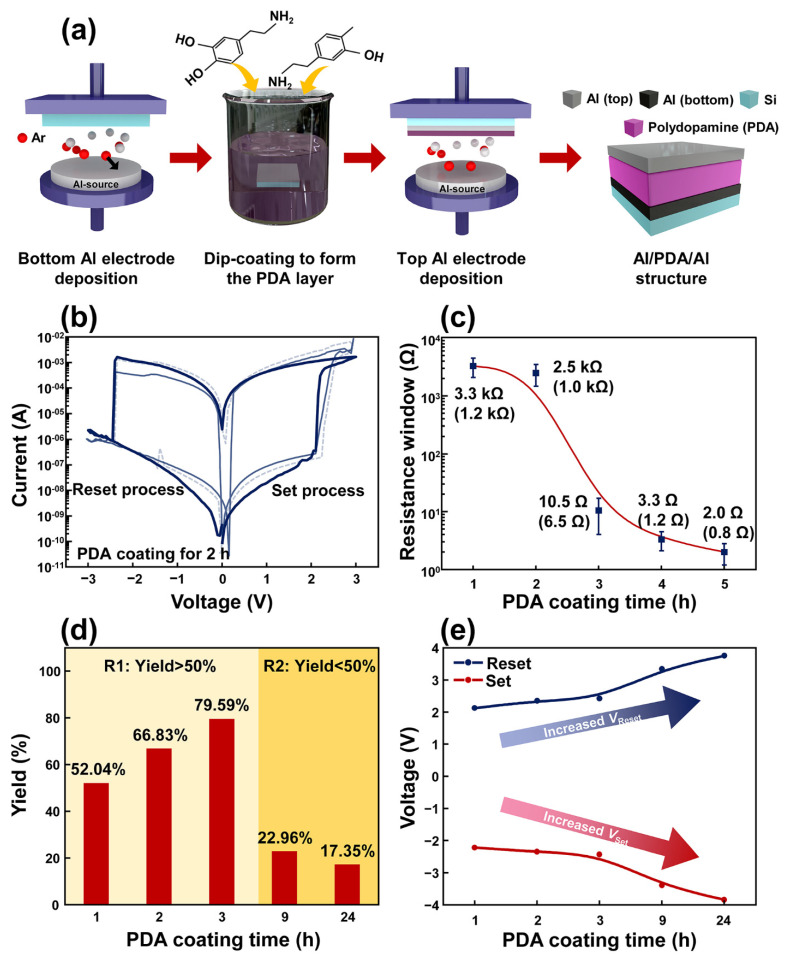
(**a**) Schematic diagram describing the fabrication process of an Al/PDA/Al structure. (**b**) Typical I–V characteristics of the Al/PDA/Al structure coated with a PDA layer for 2 h indicating a bipolar property. (**c**) The resistance window of the Al/PDA/Al structure according to the PDA coating time and standard deviation values in parentheses. (**d**) The yield of the Al/PDA/Al structure showing resistive-switching characteristics according to the PDA’s coating time. (**e**) Trends of the *V*_Set_ and *V*_Reset_ at various PDA coating times.

The detailed fabrication process of the proposed Al/PDA/Al structure is described as shown in Figure 1a. An aluminum (Al) bottom electrode was deposited on a silicon substrate by RF magnetron sputtering with an RF power of 150 W for 30 min. To form the array pattern, a shadow mask was adopted during sputtering and its optical camera image and optical microscope image can be observed in Figure S1. After the deposition of the bottom Al electrode, the PDA layer was coated on the bottom Al electrode by dip-coating accompanied by ultrasonication procedures. The pH concentration of the PDA coating solution was maintained at pH 8.3, which is known to be suitable for polymerization of the dopamine. After the dip-coating process, the fabricated PDA/Al/silicon structure was dried at room temperature for 24 h. Then, an Al top electrode was deposited by RF magnetron sputtering. As a result, the Al/PDA/Al structure was fabricated.

Figure 1b depicts the resistive switching characteristic of the fabricated Al/PDA/Al structure coated with PDA for 2 h. The applied voltage sweep was 0 V → 3 V → 0 V → −3 V → 0 V with a compliance current of 10 mA. The voltage was applied to the top electrode, and the bottom electrode served as a ground, respectively. While applying a forward-biased sweep, the current was increased dramatically at a voltage bias of 2.1 V. An abrupt change in the resistance of the Al/PDA/Al structure from a high resistance state (HRS) to low a resistance state (LRS) was observed, which is known as the “set state”. In contrast, when a reverse-biased voltage was applied to the fabricated structure, the current decreased dramatically at a bias voltage of −2.3 V, which is called the “reset state”. In Figure S2, the resistive switching of the Al/PDA/Al with a PDA layer coated for 1, 3, 9, and 24 h can be checked. Moreover, the fabricated Al/PDA/Al structure showed electroforming-free resistive switching characteristics. When the coating time for the PDA layer was increased, the resistance value of the Al/PDA/Al structure decreased, as shown in Figure 1c. The highest resistance window was observed at the Al/PDA/Al structure with a PDA coating time of 1 h, and its value and standard deviation were 3.3 kΩ and 1.2 kΩ, respectively. The high variation of 36.36% was due to the uneven surface of the PDA layer. Although the Al/PDA/Al structure showed large variation, the resistance window obviously showed the trend according to PDA coating times, as shown in Figure 1c. The variation also can be confirmed in I–V characteristics in Figure 1b.

To use Al/PDA/Al structures as resistive random-access memory devices, the on/off ratio (resistance window) should be also considered. When the coating time of the PDA exceeded 3 h, the on/off ratio of the Al/PDA/Al structure decreased to 3.2 Ω or less. Although the Al/PDA/Al structure with PDA coating times greater than 3 h showed resistance-switching characteristic, a small on/off ratio can disturb the utilization of this structure. The Al/PDA/Al structures with PDA coating times of 3 h or less are, therefore, suitable structures for the sufficient on/off ratio of RRAM devices. The number of structures, showing resistive switching, increased before 3 h of PDA coating (Figure 1d). After 3 h of PDA coating, the occurrence of resistive switching sharply decreased in Al/PDA/Al structures. The Al/PDA/Al structures coated with PDA for 3 h or less were, therefore, regarded as the suitable time period to fabricate random-access memory devices. The detailed count numbers can be checked in Table S1. In Figure 1e, the bias voltages for inducing the resistive switching were compared according to the PDA’s coating time, and the result revealed the Al/PDA/Al structure with the PDA coating for 2 h was the optimal time for PDA-based RRAM devices. When the PDA’s coating time increased, the bias voltage for inducing resistive switching of the fabricated structure also increased. After PDA coatings for more than 3 h, a voltage greater than 3 V was required to induce resistive switching in the Al/PDA/Al structure. To reduce the power consumption of the random access-memory devices, an Al/PDA/Al structure coated with a PDA coating time of less than 3 h was deemed suitable. Considering these results, the Al/PDA/Al structure coated with PDA for 2 h was selected because it demonstrated an appropriate on/off ratio, high yield, and low-operation voltage.

**Figure 2:**
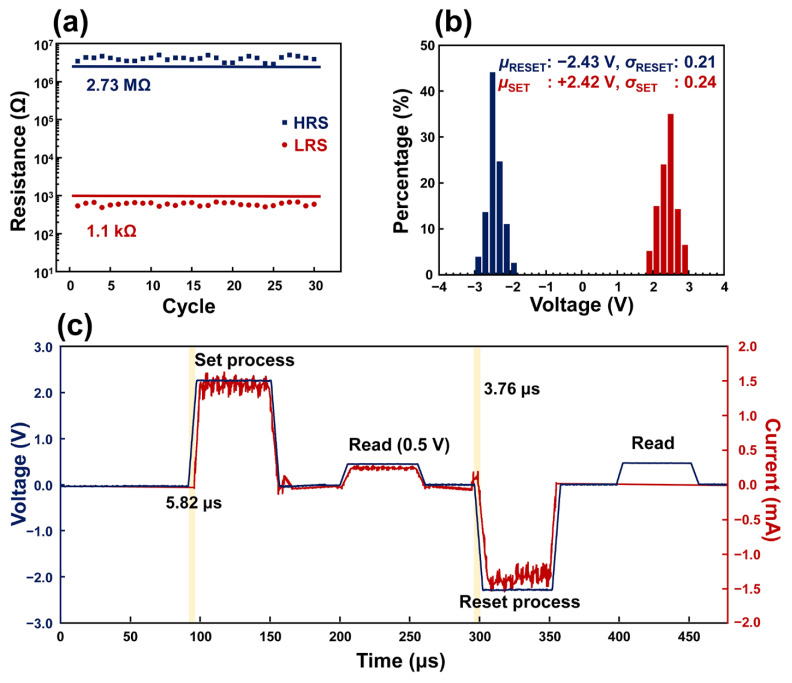
(**a**) Endurance characteristics of the Al/PDA/Al structure coated with a PDA layer for 2 h. (**b**) Percentage distribution of *V*_Set_ and *V*_Reset_ in 196 (14 × 14) Al/PDA/Al structures coated with a PDA layer for 2 h, and their mean values and standard deviations. (**c**) Typical pulse response of Al/PDA/Al structure with order of set, read, reset, and read pulses.

Figure 2 depicts the result of an investigation for resistive switching conducted with the Al/PDA/Al structure coated with a PDA layer coated for 2 h. In Figure 2a, the distribution of the resistance was investigated for 30 cycles of resistive switching. In the HRS state, the value of the resistance exceeded 2.73 MΩ, and the value of the resistance in the LRS state was less than 1.1 kΩ. The differences in the resistance of the HRS and LRS states correspond with the resistance window, as shown in Figure 1c. In addition, the Al/PDA/Al structure coated with a PDA layer for 2 h showed uniform critical voltages, which caused the set-and-reset process. The averaged voltage value (*μ*_RESET_), which caused the reset process, was −2.43 V and its standard deviation (*σ*_RESET_) corresponded to 0.21. For the set process, the averaged voltage value (*μ*_SET_) was 2.42 V and its standard deviation (*σ*_SET_) was calculated as a value of 0.24 as shown in Figure 2b. The electrical pulses were applied in order of set, read, reset, and read as shown in Figure 2c. The pulse widths were fixed to 50 μs. The set and reset response times of Al/PDA/Al structure recorded 5.82 μs and 3.76 μs, respectively. The read pulse after the set pulse demonstrated that the Al/PDA/Al structure was in the LRS state because the current (red) increased as the amplitude of the pulse increased. On the other hand, the read pulse after the reset pulse revealed that the Al/PDA/Al structure converted its state from the LRS to HRS state. Based on these results, the fabricated Al/PDA/Al structure demonstrated its excellent applicability by operating with a programmable pulse, which is commonly used in conventional electronics.

**Figure 3:**
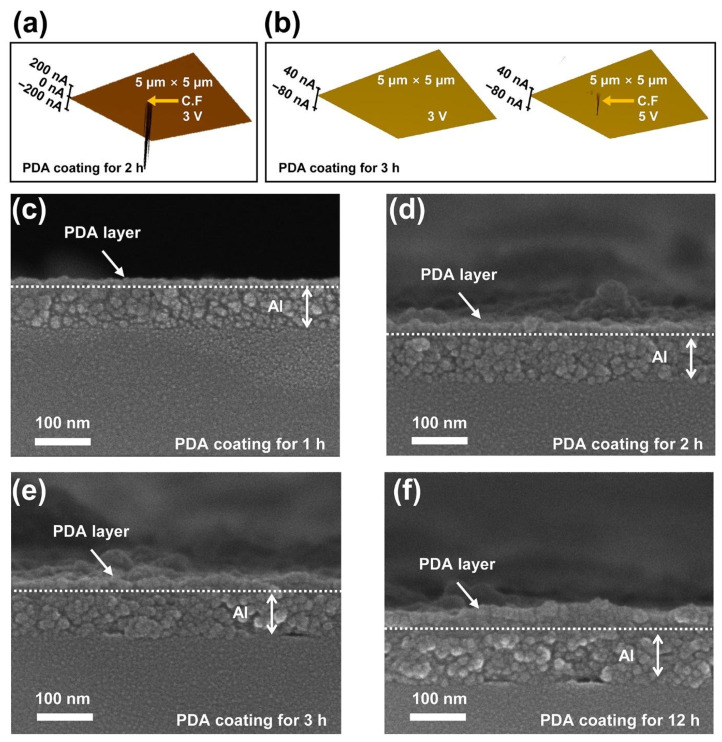
The measured conductive filament in the PDA/Al structure coated with a PDA layer for (**a**) 2 h and (**b**) 3 h with the aid of the C-AFM. The cross-section images of the PDA/Al structure coated with a PDA layer for (**c**) 1 h, (**d**) 2 h, (**e**) 3 h, and (**f**) 12 h, as observed with a scanning electron microscope.

Figure 3a,b depict surface images obtained by conductive atomic force microscopy (C-AFM) to confirm the distribution of the conductive filament (C.F) formed at the PDA layer when the PDA/Al structures coated with PDA layers for 2 and 3 h were in the HRS state. The size of the investigated area was 5 μm × 5 μm, respectively. When a bias voltage of 3 V was applied to each PDA/Al structure, conductive filaments were observed only in the PDA/Al structure coated with a PDA layer for 2 h. On the other hand, the absence of a conductive filament in the PDA/Al structure coated with a PDA layer for 3 h indicated that resistive switching did not occur. When a bias voltage of 5 V was applied to the PDA/Al structure coated with a PDA layer for 3 h, conductive filaments can be observed, as shown in Figure 3b. These results followed a trend similar to that of the *V*_Set_ described in Figure 1e. This increment in the voltage required to induce resistive switching can be explained by the increased thickness of the PDA layer due to a longer PDA coating time. Prior to formation of a conductive filament, the fabricated structure was in the HRS state because the PDA layer acted as an insulating layer. When a sufficient electric field is applied to a PDA insulator layer, soft breakdowns can occur in the PDA insulator layer, resulting in the formation of conductive filaments. Because an electric field is affected by both voltage and distance, the thickness of a PDA layer can affect the amplitudes of *V*_Set_ and *V*_Reset_, which induce resistive switching. To apply the same electric field to the Al/PDA/Al structure with an increased thickness of a PDA layer according to coating time, the applied bias voltage should be inevitably increased. To confirm the thickness of the PDA layer according to the coating time, the cross sections of the PDA/Al structures with different coating times were observed by scanning electron microscopy, as shown in Figure 3c–f. The trend and values for the thickness of the PDA layer according to coating time are provided in Figure S3 and Table S2, respectively. Considering these results, *V*_Set_ and *V*_Reset_ should be inevitably increased to apply the same electric field to the Al/PDA/Al structure for inducing resistance switching due to the increased thickness of the PDA layer as the coating time increases.

When the surface of a bare Al electrode was deposited on a silicon wafer, a high-intensity in the bond between aluminum and oxygen was observed by XPS analyses due to the nature of the aluminum, which possesses a high affinity for oxygen, as shown in Figure S4a. The measured intensity of the Al 2p peak was 32.75%. When the PDA was coated for 1 h onto the aluminum electrode, the intensity value of the Al 2p peak decreased to 22.70% as shown in Figure S4b. Finally, when the PDA was coated for 2 h onto the aluminum electrode, the intensity of the Al 2p peak reduced to 5.09%, as shown in Figure S4c. The high intensity of the Al 2p peak after PDA coating indicated that the coated PDA layer did not fully cover the Al electrode. This can create an electrical connection between the top electrode and bottom electrode after depositions of the top electrode on the PDA layer. However, the low intensity of the Al 2p peak implied that the coated PDA layer successfully covered the Al electrode, potentially preventing the formation of an electrical connection between the top and bottom electrodes. The yield of the Al/PDA/Al structures coated with PDA for 2 and 3 h indicates that resistive switching can be increased relative to that of the Al/PDA/Al structure coated with PDA coating for 1 h, as shown in Figure 1d. As shown in Figure S5 and S6, the reduction in the Al 2p peak can be checked against PDA coating time. After 2 h of the PDA coating, the Al 2p peak cannot be observed, as shown in Figure S6. This result corresponds to the investigation results, as shown in Figure S4c.

**Figure 4:**
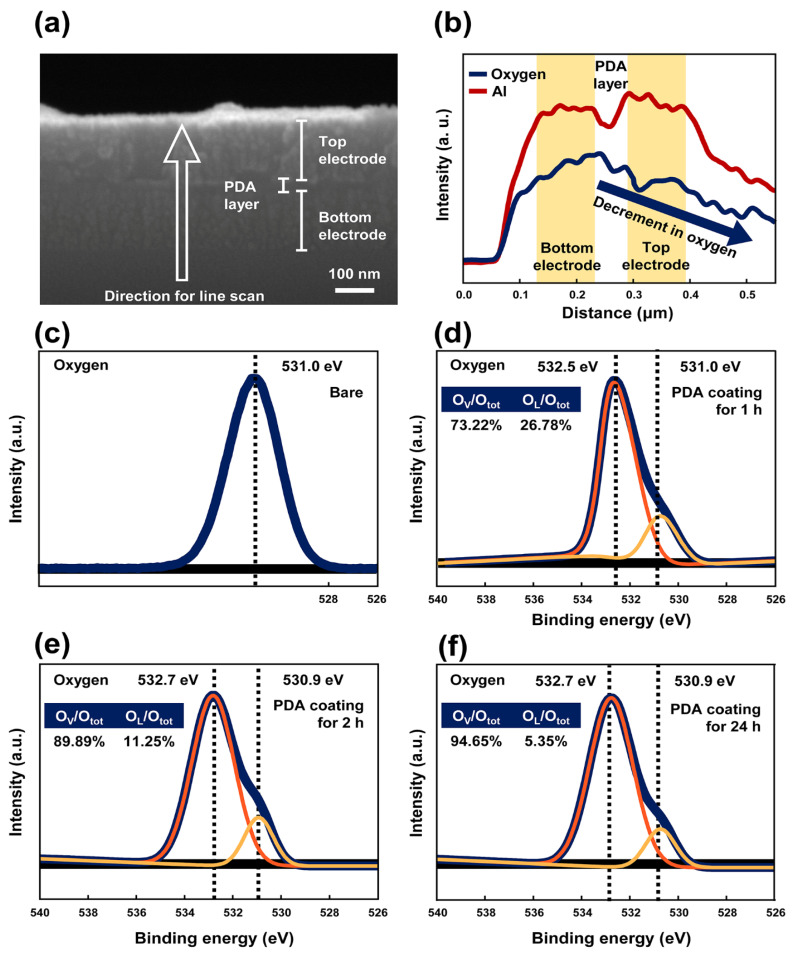
The (**a**) cross section image of Al/PDA/Al structure and (**b**) its line scan result for Al and oxygen investigated by energy dispersive X-ray spectroscopy (EDS). XPS spectra of the (**c**) O 1s in a bare Al electrode and the PDA/Al structure coated with a PDA layer coated for (**d**) 1 h, (**e**) 2 h, and (**f**) 24 h.

Figure 4a,b showed the cross-section image of Al/PDA/Al structure and its line scanning results for investigating the Al and oxygen contents in each interface. The line scan was conducted from the bottom electrode to the top electrode. The oxygen content in the bottom electrode was higher than that in the top electrode, and the oxygen content gradually decreased when the scanner investigated the top electrode compared to the bottom electrode. The highest oxygen content was recorded between the bottom electrode and the PDA layer. These results reveal that the probability for the formation of an Al_2_O_3_ layer between the bottom electrode, and the PDA layer is higher than that between the top electrode and PDA layer. These results reveal that the Al_2_O_3_ layer is dominantly formed between the bottom electrode and the PDA layer and not the location between the top electrode and the PDA layer.

The intensity of aluminum-oxygen bond also increased, as shown in Figure S4a–c, indicating that an Al_2_O_3_ layer was naturally formed on the surface of the bottom Al electrode. This can be attributed to the presence in the PDA of a catechol group, which can generate a superoxide as a byproduct of an autoxidation [38,39,40]. The generated superoxide can be regarded as an oxidant and possesses high reactivity due to its unstable state. As a result, the bond between the generated superoxide and aluminum can be easily formed, and the generated superoxide can contribute to the formation of oxygen vacancies due to its unstable state. To confirm the formation of oxygen vacancies, the O 1s peak was analyzed by the XPS. In Figure 4c, the binding energy of O 1s in the Al electrode deposited on the silicon wafer was 531.0 eV. After PDA coating, the O 1s peak was changed as shown in Figure 4d–f. The XPS analysis results showed two peak values with binding energies of 532.5~532.7 eV and 530.09~531.0 eV, which corresponded to the binding energy of the oxygen vacancy and lattice oxygen, respectively. The ratio of the oxygen vacancy to the total area of the O 1s increased with PDA coating time. Finally, the ratio of the oxygen vacancy to the total area of the O 1s reached 94.65% after PDA coating for 24 h. The occurrence of the multiple oxygen vacancies in an insulator layer can create multiple uncontrollable leakage paths, resulting in poor resistive switching at a low on/off ratio. As a result, low on/off ratios were observed with the Al/PDA/Al structures coated with a PDA layer for more than 9 h, as shown in Figure 2b. Moreover, these high contents of the oxygen vacancies in the initial state of PDA/Al structure might affect its electroforming-free resistive-switching characteristic, which is a process for creating oxygen vacancies. Between the top Al electrode and the PDA layer, there were a few catechol groups because the catechol groups were autoxidized during drying process. Hence, the quinones appeared on the surface between the top Al electrode and the PDA layer, resulting in a few formations of Al_2_O_3_ layers between the top Al electrode and the PDA layer.

**Figure 5:**
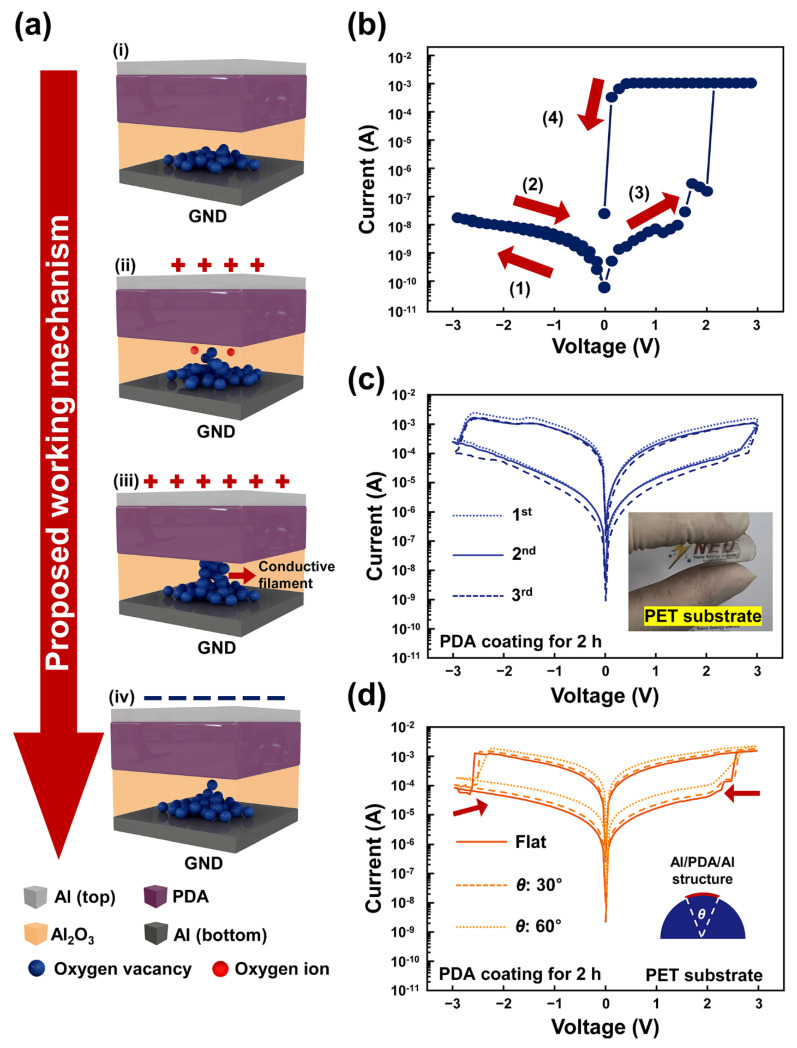
(**a**) The proposed resistive-switching mechanism of the Al/PDA/Al structure. (**i**) Initial state. (**ii**) The state applying the forward-bias voltage. (**iii**) Creation of a conductive filament due to the sufficient forward-bias voltage. (**iv**) Rupture of the conductive filament due to reverse-bias voltage. (**b**) The I-V characteristics of the proposed Al/PDA/Al structure with an applied voltage sweep (0 V → −3 V → 0 V → 3 V → 0 V). (**c**) The I-V characteristics measured with PDA-based RRAM using PET substrate and (**d**) its I-V characteristics in flat condition and bending conditions of 30° and 60°.

These results show that oxygen vacancies play a key role in the formation of conductive filaments in an Al/PDA/Al structure. A working mechanism for resistive switching in an Al/PDA/Al structure is proposed, as shown in Figure 5a. In the initial state (i), the oxygen vacancies are near the bottom electrode, based on the XPS spectra, as shown in Figure 4e,f. When the sufficient forward-bias voltage is applied to the top Al electrode, the PDA insulator layer breaks down, and oxygen ions in the naturally formed Al_2_O_3_ layer between the bottom Al electrode and the PDA layer migrate toward the PDA layer, as shown in Figure 5a(ii). Due to the electric field, the oxygen ions can be escaped from the oxygen lattice. After the migration of the oxygen ions, conductive filaments form due to the oxygen vacancies, as shown in Figure 5a(iii). Electrons can, therefore, flow due to the conductive filaments formed between the PDA layer and the bottom Al electrode. Then, resistive switching occurs and the Al/PDA/Al structure is in the LRS state. When the reverse-bias voltage is applied to the Al/PDA/Al structure, the formed conductive filaments are ruptured as shown in Figure 5a(iv). As a result, resistive switching occurs from the LRS state to the HRS state.

To confirm this proposed resistive-switching mechanism, the voltage sweep changed from 0 V → 3 V → 0 V → −3 V → 0 V to 0 V → −3 V → 0 V → 3 V → 0 V, as shown in Figure 5b. Because aluminum was utilized for both the top and bottom electrodes, the Al/PDA/Al structure is symmetrical from top to bottom in the absence of a naturally formed Al_2_O_3_ layer between PDA and bottom Al electrode. The resistive switching should, therefore, occur in the reverse direction when the voltage sweep was applied to the top electrode in the initial state. However, the set process was not observed when the reverse-bias voltage was applied to the top electrode at the initial state. When the forward-bias voltage was applied to the top electrode, the set process was observed, as shown in Figure 5b. This can be attributed to the fabrication process of the Al/PDA/Al structure. When the dip-coating was conducted in order to fabricate the PDA layer on the bottom Al electrode, the bottom Al electrode and PDA coating solution were in contact. Compared to the top Al electrode, this contact can provide more opportunities for PDA and bottom Al electrode to form an Al_2_O_3_ layer. Due to the non-aligned molecular arrangement of the polymers, an Al_2_O_3_ layer can form between the PDA layer and the top Al electrode. However, because the bottom Al electrode was in contact during polymerization, the opportunity to form an Al_2_O_3_ layer between bottom Al electrode and PDA layer is greater than that between the top Al electrode and PDA layer. Hence, the Al_2_O_3_ layer is dominantly formed between the bottom Al electrode and the PDA/Al structure. The PDA/Al structure was dried for 24 h in room temperature, which indicated that the catechol group in the PDA layer was already autoxidized. Hence, it is difficult for the top electrode to come into contact with the catechol group in the PDA layer. As a result, the resistive switching can be observed only when a forward-bias voltage was applied at the top Al electrode. Considering these results, the resistive switching characteristic observed at the Al/PDA/Al structure can be explained by the repetitive formation and rupture of the conductive filament in naturally formed Al_2_O_3_ layers between the PDA layer and the bottom Al electrode. These results reveal that the PDA attributes the formation of the Al_2_O_3_ between a bottom Al electrode and an PDA layer, which has a key role in the resistive switching of Al/PDA/Al structures.

As an application, a PDA-based RRAM was fabricated using polyethylene terephthalate (PET) substrate to demonstrate the potential of the proposed structure as flexible RRAM devices. In Figure 5c, the I–V characteristics of PDA-based RRAMs using PET substrate were revealed. By varying the substrate material from Si to PET, the on/off ratio of the proposed structure decreased. This difference in on/off ratio results from the crystal structure of Si and PET. With general knowledge, the Si wafer possesses a crystal structure when polymers show irregular crystal structures. Hence, the occurrence of conductive filament with the proposed structure using PET substrate becomes more uncontrollable compared to Al/PDA/Al structures using Si substrate. However, the PET substrate can provide flexibility to its structure, as shown in the inset of Figure 5c. Although various flexible and wearable sensors have been proposed, few flexible data storage devices have been proposed. Compared to inorganic materials, organic materials possess a flexibility, which can be adopted for flexible and wearable devices. The I-V characteristics of Al/PDA/Al structures using PET substrates were investigated with flat condition and bending conditions of 30° and 60°, respectively. When the bending angle increased, the set-and-reset voltages and the resistance value of the proposed structure decreased, as shown in Figure 5d. These results resulted from the mechanical deformation of the insulator layer. When the PET substrate was in a bending state, the length of the active area increased due to the increased PETs by bending strain. Figure S7a,b show the surface of PDA/Al structures before and after bending, investigated by AFM, and Figure S7c,d showed the surface of PDA/Al structures before and after bending at 60°, investigated by SEM. After bending, the surface of the PDA/Al structure was deformed. Although the same voltage was applied for resistive switching, some parts of PDA/Al structure went up and these parts affected the I-V characteristics because the electric field between PDA and top electrode increased by decreasing distances between the PDA layer and the top electrode. However, resistive switching can be observed with the Al/PDA/Al structure using PET substrates despite its bended state. These results revealed the great potential of the proposed Al/PDA/Al structure as a flexible memory device.

## 4. Conclusions

In summary, the Al/PDA/Al structure was fabricated and its resistive-switching characteristic was investigated. The optimized coating time of the PDA was 2 h by achieving the best performance in various parameters of the Al/PDA/Al structure, including its resistance window, yield, and operation voltage. The on/off ratio of the Al/PDA/Al structure coated with a PDA layer 2 h was 2.48 × 10^3^. The averaged values of the voltages for the set-and-reset process of the Al/PDA/Al structure coated with a PDA layer for 2 h were 2.42 V and −2.43 V with standard deviations of 0.21 and 0.24. XPS spectra results for Al 2p revealed that an Al_2_O_3_ layer formed between the PDA layer and the bottom Al electrode. In terms of the O 1s peak, an increment in the number of oxygen vacancies was observed as PDA’s coating time increased. Moreover, a conductive filament formed between the PDA layer and the bottom Al electrode. These results revealed its potential as a predicted working mechanism for resistive switching in a proposed Al/PDA/Al structure based on the oxygen vacancy-induced conductive filament in the Al_2_O_3_ layer. To confirm the proposed working mechanism, a reverse-voltage sweep was applied to the Al/PDA/Al structure, and the set process was only observed at forward-bias voltage. As an application, the flexible RRAM device was fabricated using the PET substrate. Although the proposed structure was bent at 30° and 60°, resistive switching can be observed. Considering these results, the resultant resistive-switching characteristics of the proposed Al/PDA/Al structure improve our understanding of PDAs and PDA-based organic RRAMs for flexible and wearable RRAM devices.

## Data Availability

Not applicable.

## Supplementary Materials

The following supporting information can be downloaded at: https://www.mdpi.com/article/10.3390/polym14152995/s1, Figure S1: The image of the utilized shadow mask. (a) Optical camera image of the utilized shadow mask. (b) Optical microscope image of the utilized shadow mask. Figure S2: I-V characteristics of the Al/PDA/Al structure according to PDA coating time. Figure S3: Thickness of the PDA layer according to PDA coating time. Figure S4: XPS spectra of (a) Al 2p in a bare Al electrode and a PDA/Al structure with a PDA layer coated for (b) 1 h and (c) 2 h, respectively. Figure S5: XPS spectra of the PDA/Al structure according to PDA coating time. Figure S6: Enlarged XPS spectra of the Al and PDA/Al structure coated with a PDA layer coated for 1 h and 2 h. Figure S7: AFM images of (a) PET substrate-based PDA/Al structure with flat condition and (b) bending condition (bending angle: 60°). SEM image of (c) PET substrate-based PDA/Al structure with flat condition and (d) bending condition (bending angle: 60°). Table S1: Count of resistive switching (RS) and non-RS characteristics. Table S2: Thickness of the PDA layer according to PDA coating time.
