# Supplementary material for: Unraveling the Role of Polydopamines in Resistive Switching in Al/Polydopamine/Al Structure for Organic Resistive Random-Access Memory
Source: Polymers (Basel). 2022 Jul 24;14(15):2995. doi: 10.3390/polym14152995 (PMC9332170; doi:10.3390/polym14152995)
Supplement: Supplementary file 1 [file polymers-14-02995-s001.zip › polymers-1798171-supplementary.pdf]

Supplementary Materials: Unraveling the Role of Polydopamines in Resistive Switching in Al/Polydopamine/Al Structure for Organic Resistive Random-Access Memory

Jonghyeon Yun and Daewon Kim

1. The image of the utilized shadow mask.

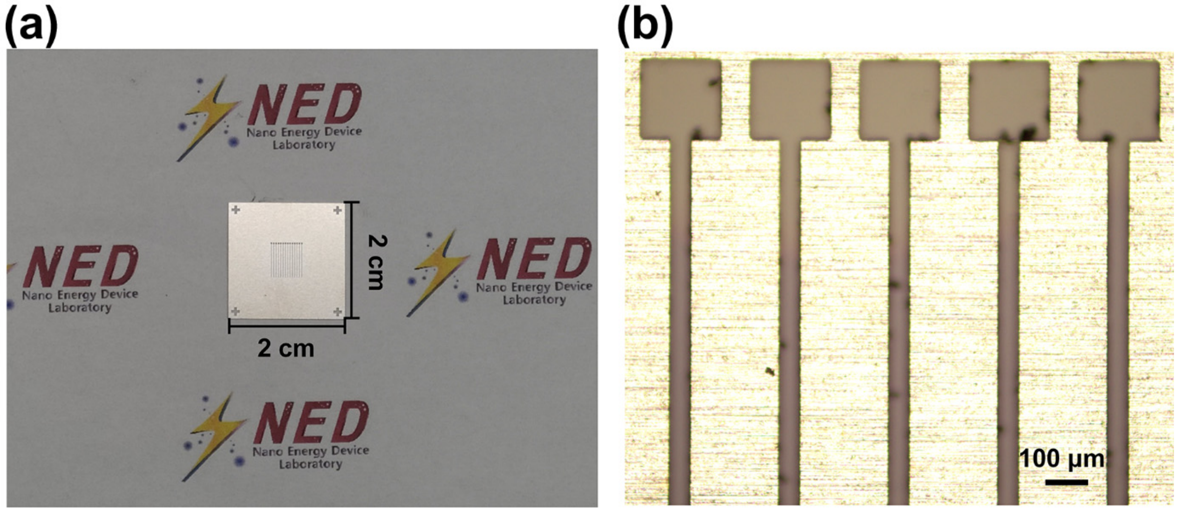

Figure S1. (a) Optical camera image of the utilized shadow mask. (b) Optical microscope image of the utilized shadow mask.

2. *I*-*V* characteristics of the Al/PDA/Al structure according to PDA coating time.

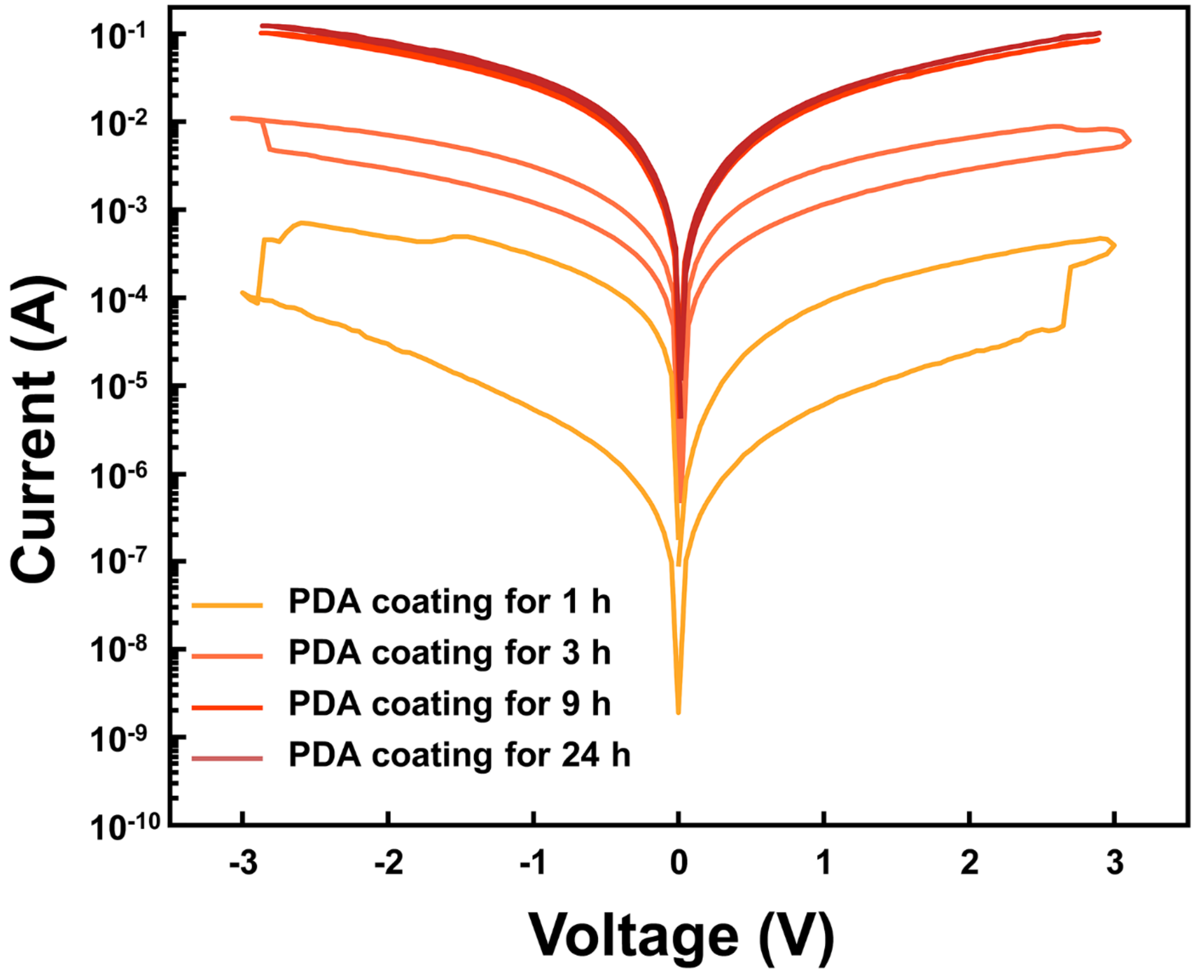

Figure S2. *I*-*V* characteristics of the Al/PDA/Al structure showing resistive switching according to PDA coating time.

3. Thickness of the PDA layer according to PDA coating time.

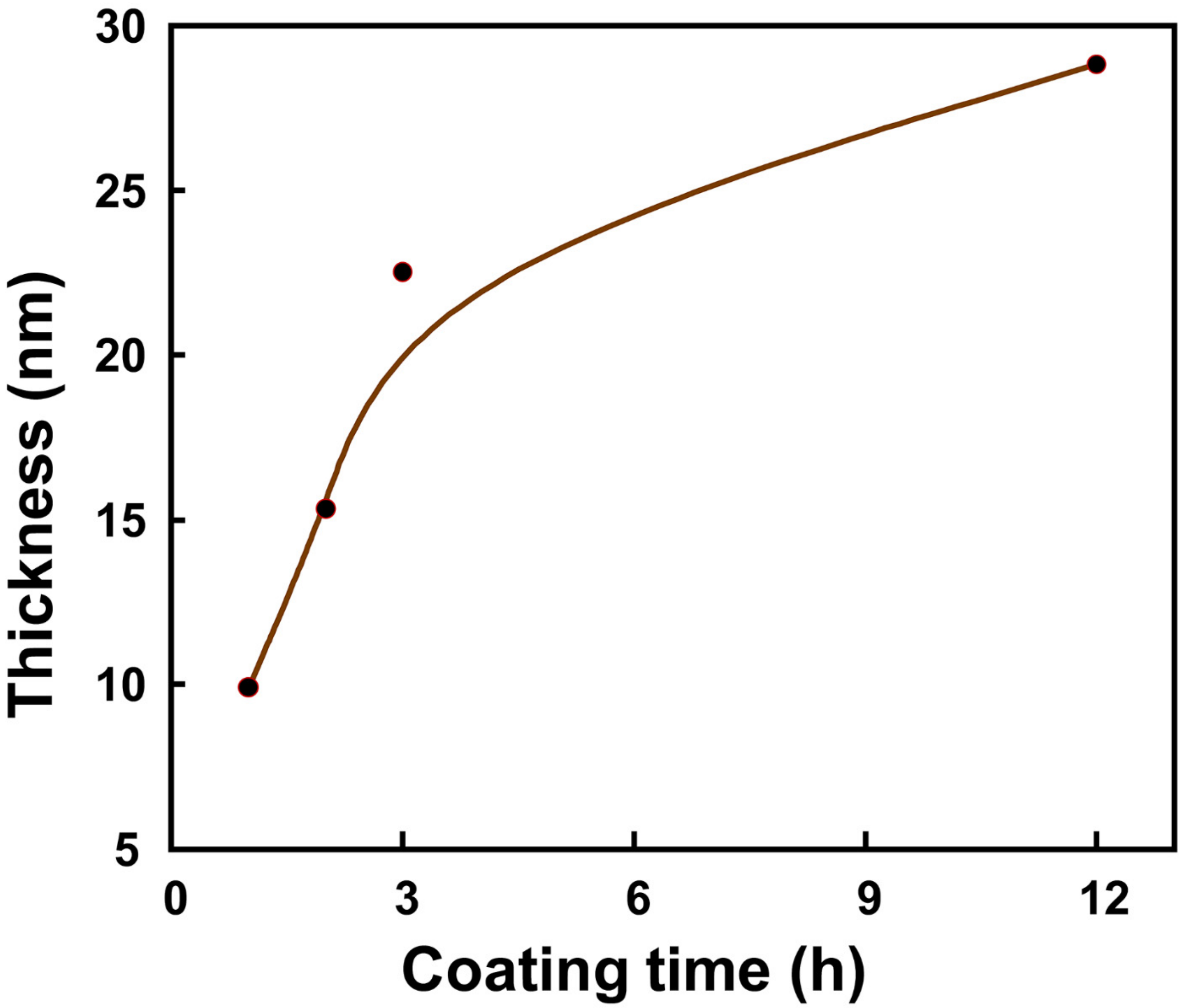

Figure S3. Thickness of the PDA layer according to PDA coating time.

4. XPS spectra of the Al and PDA/Al structure coated with a PDA layer coated for 1 h and 2 h.

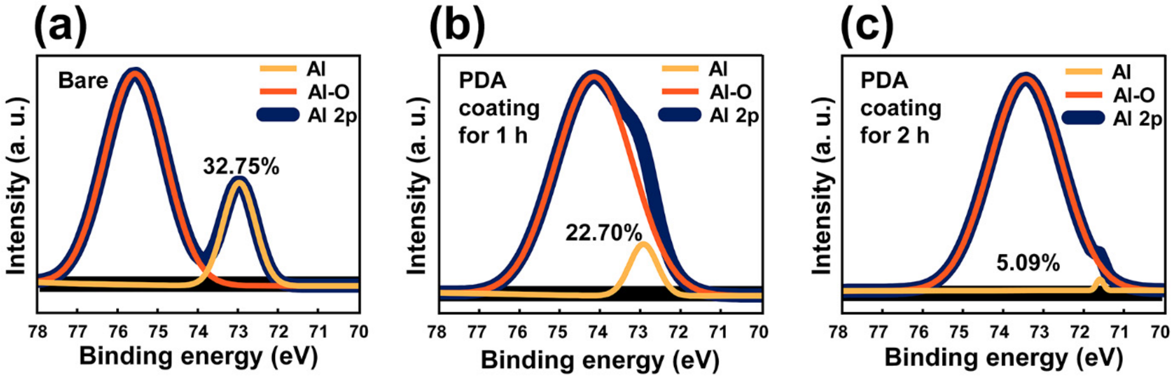

Figure S4. XPS spectra of (a) Al 2p in a bare Al electrode and a PDA/Al structure with a PDA layer coated for (b) 1 h and (c) 2 h, respectively.

5. XPS spectra of the PDA/Al structure according to PDA coating time.

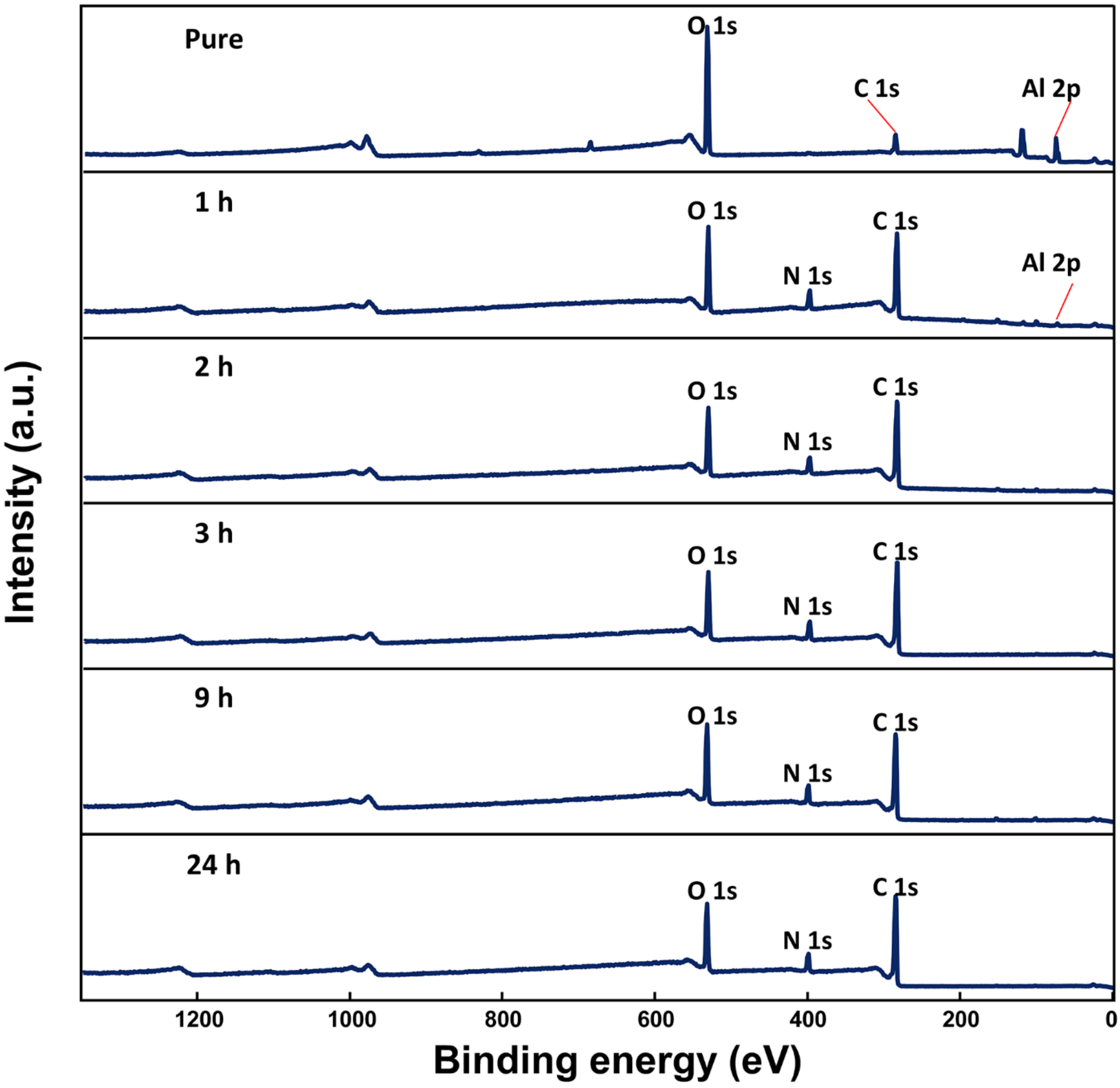

Figure S5. XPS spectra of the Al (2p), C (1s), N (1s), and O (1s) in the PDA/Al structure according to PDA coating time.

6. Enlarged XPS spectra of the Al and PDA/Al structure coated with a PDA layer coated for 1 h and 2 h.

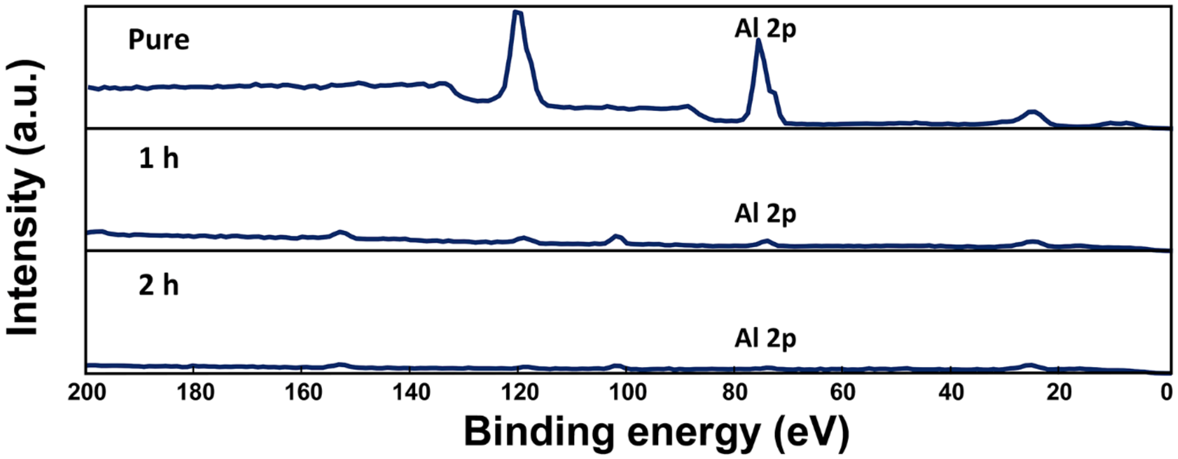

Figure S6. Enlarged XPS spectra of the Al (2p) in the PDA/Al structure according to PDA coating time.

7. AFM and SEM images of PET substrate-based PDA/Al structure.

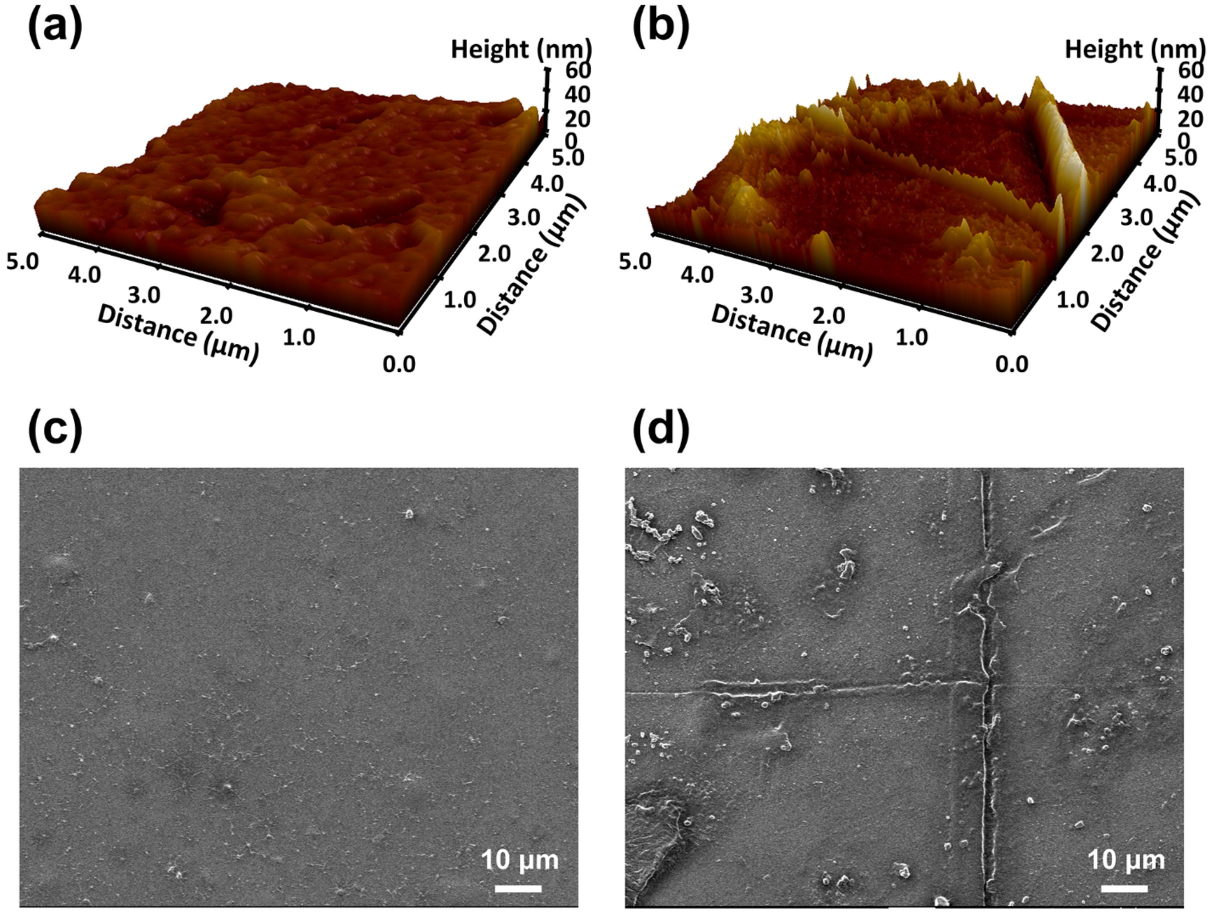

Figure S7. AFM images of (a) PET substrate-based PDA/Al structure with flat condition and (b) bending condition (bending angle: 60°). SEM image of (c) PET substrate-based PDA/Al structure with flat condition and (d) bending condition (bending angle: 60°).

8. Count of resistive switching (RS) and non-RS characteristics

Table S1. Count times of RS and non-RS characteristics

Coating time (h)	1	2	3	9	24
RS characteristics	102	131	156	45	34
Non-RS characteristics	94	65	40	151	162
Total	196	196	196	196	196
Yield (%)	52.04	66.83	79.59	22.96	17.35

9. Thickness of the PDA layer according to PDA coating time.

Table S2. Thickness of the PDA layer according to PDA coating time in SEM image.

Coating time (h)	Thickness (nm)
1	9.91
2	15.32
3	22.52
12	28.83
